# Effects of purifiers on the airborne transmission of droplets inside a bus

**DOI:** 10.1063/5.0081230

**Published:** 2022-01-18

**Authors:** Yafeng Yang, Yiping Wang, Linli Tian, Chuqi Su, Zhixin Chen, Yuanyi Huang

**Affiliations:** 1 Hubei Key Laboratory of Advanced Technology for Automotive Components, Wuhan University of Technology, Wuhan, Hubei 430070, China; 2 Hubei Collaborative Innovation Center for Automotive Components Technology, Wuhan University of Technology, Wuhan, Hubei 430070, China; 3 SAIC GM Wuling Automobile Co., Ltd, Liuzhou, Guangxi 545000, China

## Abstract

During an airborne infectious disease outbreak, bus passengers can be easily infected by the dispersion of exhaled droplets from an infected passenger. Therefore, measures to control the transport of droplets are necessary, such as a mask or purifier. The current research examined aerosol transport in a bus with air-conditioning. To determine the dispersion path, deposition distribution, and droplet escape time, the computational fluid dynamics were used to predict the flow field and the dispersion of droplets considering the effects of droplet size, location of the infected person, and purifier type. In addition, based on the viability and the number of virus particles in a droplet, the total number of virus particles inhaled by passengers over a 4-h journey was obtained by the superposition method. The Wells–Riley equation was then used to assess the infection risk of the passengers in the bus cabin. The results showed that droplets with a size of 1–20 *μ*m have essentially the same deposition characteristics, and the location of the infected passenger affects the distribution of droplets' transport and the effectiveness of a purifier in removing droplets. A purifier can effectively remove droplets from passengers' coughs and reduce the infection risk of passengers. The performance of the smaller purifiers is not as stable as that of the larger purifiers, and the performance is influenced by the airflow structure where the infected passenger is located.

## I. INTRODUCTION

Since the severe acute respiratory syndrome (SARS) epidemic swept across the world in 2003, respiratory infectious disease outbreaks, such as the bird flu in 2005, swine influenza in 2009, and new coronavirus pneumonia (COVID-19) in 2019 have erupted one after the other. The COVID-19 epidemic has caused serious public health problems and enormous economic losses.^1^ Generally, the transmission of respiratory infectious diseases occurs via three routes, namely, contact, airborne, and fomite.^2^ With regard to airborne transmission, the respiratory droplets and droplet nuclei expelled by an infected individual can travel up to tens of meters and be inhaled by a susceptible individual, leading to the risk of infection. Therefore, airborne transmission has increased the difficulty of epidemic prevention and control. Compared to outdoor environments, airborne transmission is more likely to occur in enclosed spaces, such as buildings,^3–5^ airplanes,^6–8^ trains,^9^ and buses.^10,11^ Among these spaces, the risk of infection via airborne transmission may be highest in buses because of high occupant density, poor ventilation configuration, and long exposure time.

Many public health emergencies have shown that infected bus passengers can transmit infectious diseases.^12,13^ For example, many people were infected with H1N1 on an Australian island in 2009, and contact tracing revealed that 12 bus passengers were infected by an infected individual on a bus who had returned to the island from Canberra. The disease then spread rapidly across the island.^14^ During the outbreak of the COVID-19 epidemic, a study by Shen *et al.* showed that 24 of 68 people on a long-distance bus ride became infected. The investigation linked buses with a high transmission rate.^10^ Another study reported that 12 passengers were infected by a single individual on two buses in a single day.^11^ In addition, a study by Jones *et al.*^15^ reported two episodes of potential COVID-19 transmission from infected van drivers to passengers despite masking and physical distancing.

Those previous studies indicated that the bus ventilation systems are typically incapable of controlling virus transmission. Clearly, improving the ventilation systems could reduce virus transmission, but implementing such improvements on buses would be expensive and laborious. However, the use of purifiers would be more convenient and economical. Air purifiers have been widely used to remove suspended virus particles in various enclosed environments during the COVID-19. Evaluating the performance of purifiers in terms of droplet removal and virus transmission control is, therefore, warranted.

Considerable recent attention has been focused on the effect of purifiers in controlling virus transmission. Burgmann *et al.*^16^ carried out a numerical and experimental investigation of the role of purifiers in reducing the concentration of droplets in classrooms. Their study showed that the effectiveness of the purifiers primarily depends on their position relative to the infected person. He *et al.*^5^ numerically investigated the effect of box fan air purifiers on droplet dispersion and removal in a poorly ventilated classroom. The results of their study suggested that the box fan purifiers are effectively at removing droplets and significantly reduce the risk of airborne infection. Moreover, the closer the purifier is located to the infected person, the more effectively it removes droplets. In addition, inappropriate purifier arrangements can pose a higher infection risk for people in specific locations. Narayanan *et al.*^17^ studied the application of purifiers in a music classroom and considered the effects of the masks, musical instruments, and injection rate. Their results showed that the use of purifiers could achieve World Health Organization standards for ventilation efficiency. However, few studies have examined the effect of purifiers on droplet dispersion and removal in an enclosed bus environment.

To fill this gap, the effectiveness of small seat-mounted and large ceiling-mounted air purifiers in removing droplets generated by the coughing of an infected person and reducing the infection risk to other passengers was investigated in current research. Generally, researchers believe that virus particles can be carried by droplets exhaled by an infected person.^18–20^ Thus, it is possible to assess the infection risk by combining information regarding the number of inhaled droplets containing an infectious agent and an appropriate risk assessment model.^21–24^ Therefore, accurate predictions of the distribution of exhaled droplets within the bus is crucial. The computational fluid dynamics (CFD) method is a powerful tool for simulating complex and coupled physical conditions quickly, accurately, and inexpensively.^25^ Specifically, CFD was employed to comprehensively study the transport characteristics of droplet released from a sing cough. Then, the number of droplets inhaled by passengers could be obtained based on the droplet distribution. The superposition method which can accumulate the number of virus inhalation by passengers from a single cough to multiple coughs is introduced to obtain the total number of virus particles inhaled by passengers during the journey. Finally, the probability of infection is predicted by the Wells–Riley equation for passengers after a 4-h bus journey. In addition, the effect of droplet size, location of the infected passenger, and purifier type were investigated. This study attempted to provide a basis and guidance for using air purifiers to eliminate virus-containing aerosolized droplets on buses.

## II. RESEARCH METHODS

As mentioned in the Introduction, assessing the infection risk of a person inside an enclosed space requires accurate prediction of the distribution of exhaled droplets. The flow field inside the bus in this study was predicted using CFD, and the Lagrangian approach was used to track droplets. Combined with Wells–Riley equations, the infection risk of passengers was then evaluated.

### A. Turbulence model

The appropriateness of the turbulence model selected will directly affect simulation accuracy and efficiency. Several researchers have applied different turbulence models to predict the airflow field in a confined space.^26,27^ The renormalization group model exhibits better overall performance and was, therefore, adopted in current research,

$$\frac{\partial }{\partial t}(\rho k)+\frac{\partial }{\partial x_{i}}\left(\rho ku_{i}\right)=\frac{\partial }{\partial x_{j}}\left(\alpha_{k}\mu_{t}\frac{\partial k}{\partial x_{j}}\right)+G_{k}+G_{b}-\rho \varepsilon -Y_{m}+S_{k},$$ (1)

$$\frac{\partial (\rho \varepsilon )}{\partial t}+\frac{\partial }{\partial x_{i}}\left(\rho \varepsilon u_{i}\right)=\frac{\partial }{\partial x_{j}}\left[\left(\alpha_{\varepsilon }\mu_{t}\right)\frac{\partial \varepsilon }{\partial x_{j}}\right]+C_{1\varepsilon }\frac{\varepsilon }{k}\left[\left(G_{k}+C_{3\varepsilon }G_{b}\right)\right]-C_{2\varepsilon }\rho \frac{\varepsilon^{2}}{k}-R_{\varepsilon }+S_{\varepsilon },$$ (2)

where $u_{i}$ is the time-averaged velocity; $\mu_{t}$ is the turbulent viscosity; ρ is the air density; t is the time; $G_{k}$ and $G_{b}$ represent the generation of turbulence kinetic energy due to the mean velocity gradients and buoyancy, respectively; $Y_{m}$ represents the contribution of the fluctuating dilatation caused by diffusion; $C_{1\varepsilon }$, $C_{2\varepsilon }$, and $C_{3\varepsilon }$ are the constants; $\alpha_{k}$ and $\alpha_{\varepsilon }$ are the turbulent Prandtl numbers for k and ε, respectively; and $S_{k}$ and $S_{\varepsilon }$ are the source terms.

### B. The Lagrangian approach

In general, methods to predict a particle's transport can be classified as Eulerian or Lagrangian approaches. The performance of the two approaches in predicting particle distribution in a closed space was compared by Zhang *et al.*^28^ Their results demonstrated that both the Eulerian and Lagrangian approach can reasonably predict the particle distribution under steady conditions, but the Lagrangian approach performs better in tracing the transit spread of droplets from sources, such as coughing and sneezing. Therefore, the Lagrangian approach was used to model the dispersion of cough droplets in the current investigation,

$$\frac{d{\vec{u}}_{p}}{dt}=\frac{18u_{a}}{\rho_{p}d_{p}{}^{2}C}\left(\vec{u}-{\vec{u}}_{p}\right)+\frac{\vec{g}\left(\rho_{p}-\rho \right)}{\rho_{p}}+{\vec{F}}_{a},$$ (3)

where *u_p_* is the particle velocity, *u* is the air velocity, *u_a_* is the molecular viscosity of air, *t* is the time, *ρ*_p_ is the particle density, *ρ* is the air density, *g* is the gravitational acceleration, *d_p_* is the particle diameter, *C* is the Cunningham correction factor, and *F_a_* represents the additional forces (per unit mass).

### C. Wells–Riley equation

To estimate the probability of indoor airborne transmission of an infectious agent, the Wells–Riley equation was proposed to estimate the infection risk due to indoor airborne transmission, considering the inhaled dose of an infectious agents in terms of the number of quanta.^29,30^ Quanta represents the hypothetical infectious dose unit defined by Wells, which indicates that if a person inhales one quanta, the probability of that person becoming infected is 1–1/e,

$$P=\frac{D}{S}=1-\text{exp}\;\left(-\frac{\mathrm{Iqp}}{Q}t\right)=1-\text{exp}\;\left(-N_{s}\right),$$ (4)

where *P* is the probability of infection for a susceptible person, *D* is the number of infection cases, *S* is the number of susceptible people, *I* is the number of infected persons, *p* is the breathing rate of a susceptible person, and *q* is the quanta generation rate. To date, a number of studies have investigated the quanta generation rate for different diseases. For example, Beggs *et al.*^31^ concluded that the quanta generation rate for tuberculosis patients ranges from 1.25/h to 30840/h. The tuberculosis quanta release rate for a secondary-infected case back-calculated by Rudnick *et al.*^19^ showed that the quanta generation rate was 79/h and 128/h for 0.1 and 0.5 air changes per hour (ACH), respectively. Miller *et al.*^32^ analyzed the data from a COVID-19 case and concluded that the rate is approximately 970/h. In addition, Hsu *et al.*^33^ reported that the cough frequency can vary from 12 to 35 times per hour. Hence, the quanta generation number is assumed to be 20 per cough (geometric mean), and an average of 15 coughs per hour was considered in the current study. For Eq. (4), parameter *t* is the total exposure time, and Q is the ventilation rate, whereas $N_{s}$ is the total number of quanta that inhaled by a susceptible passenger. All passengers were assumed to be in a relatively peaceful state, with a breathing rate of 10 L/min. As mentioned in the Introduction. Infectious agents are carried by exhaled droplets from the infected passenger. Hence, the distribution of quanta is similar to that of droplets, and it is not uniform and varies over time. According to the spatial and temporal distribution of expiratory droplets which calculated using CFD, the following equation was proposed to obtain the total number of quanta inhaled by a person over a period of time:^19^

$$N(t_{0})=cp\int_{0}^{t_{0}}v(x,t)f(t)dt,$$ (5)

where *N (t_0_)* is the total number of quanta inhaled by a susceptible person from 0 to *t_0_*, *v(x, t)* is the droplet concentration in the breathing area of a susceptible passenger, *f(t)* is the viability of the virus, usually taken as 1, and *c* is the quanta concentration in the respiratory fluid. The droplet distribution in the cabin was determined using CFD, and the user-defined-functions (UDFs) of FLUENT were used to monitor the droplet concentration in each passenger's breathing zone in real-time, after which the number of quanta inhaled by each passenger was calculated using Eq. (5). However, bus trips can last several hours or longer. Therefore, the superposition method was introduced to determine the cumulative number of quanta inhaled by passengers as a result of a single cough to multiple coughs,^34^

$$N(t)=\sum_{\text{for}\;\text{all}\;i}N_{\text{single}\;}\left(t-t_{i}\right),$$ (6)

where a single cough assumed to be started at time *t_i_*, and *N*_single_ (*t* − *t*_i_) is the quanta inhaled by passengers from *t* to *t_i_*. With the superimposition method, only the calculation of droplet transport from a single cough is needed for Lagrangian particle tracking. Finally, the Wells–Riley equation was used to predict the infection risk of passengers for the whole journey.

## III. PHYSICAL MODEL AND SIMULATION CASE SETTING

### A. Bus cabin geometry and numerical procedure

**FIG. 1.**
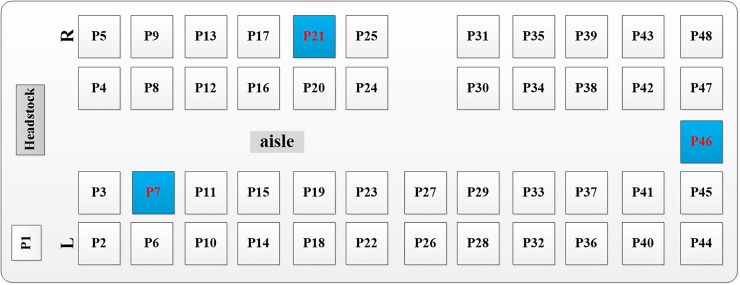
Bus seat arrangement (P1: driver, P2-P48: passengers).

**FIG. 2.**
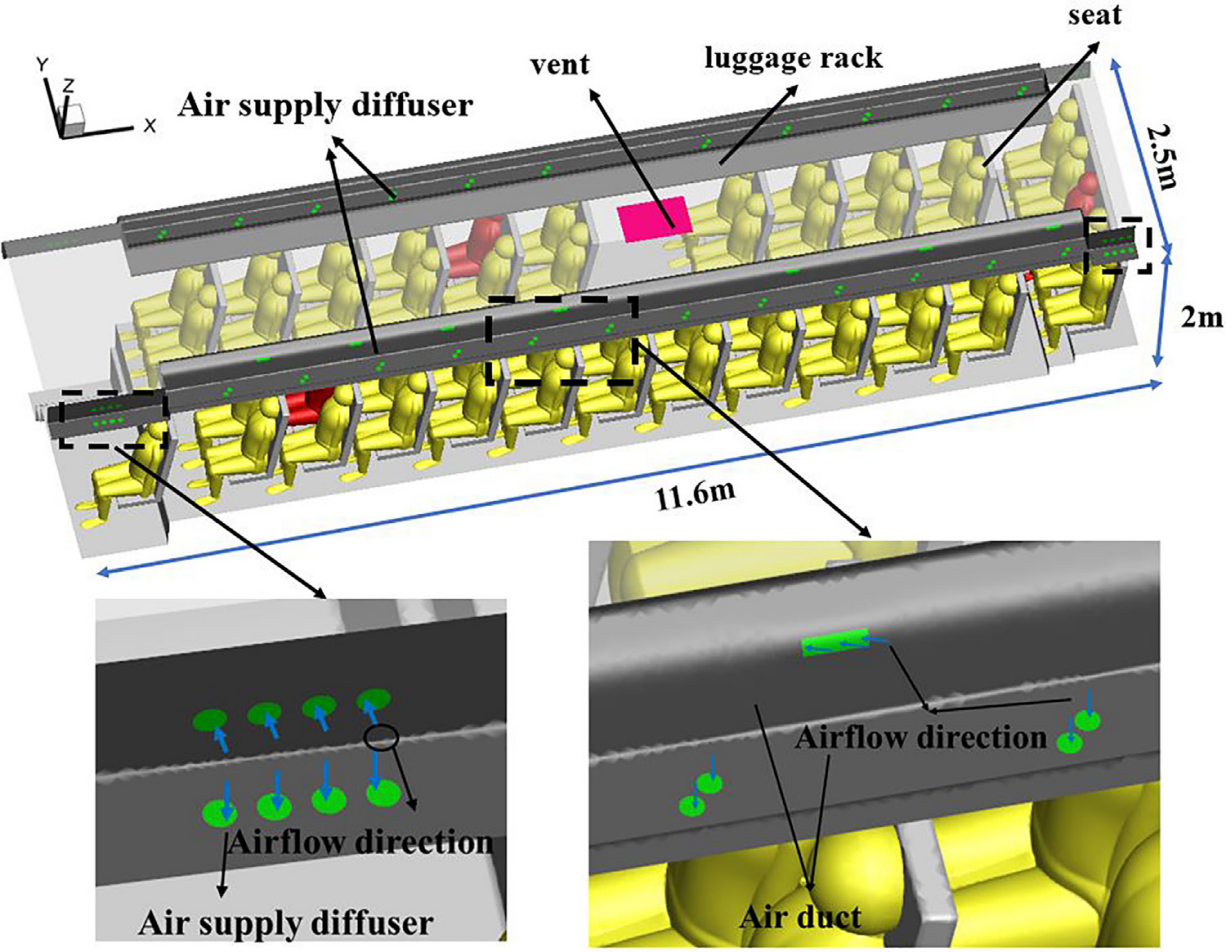
Schematic illustration of the bus.

**FIG. 3.**
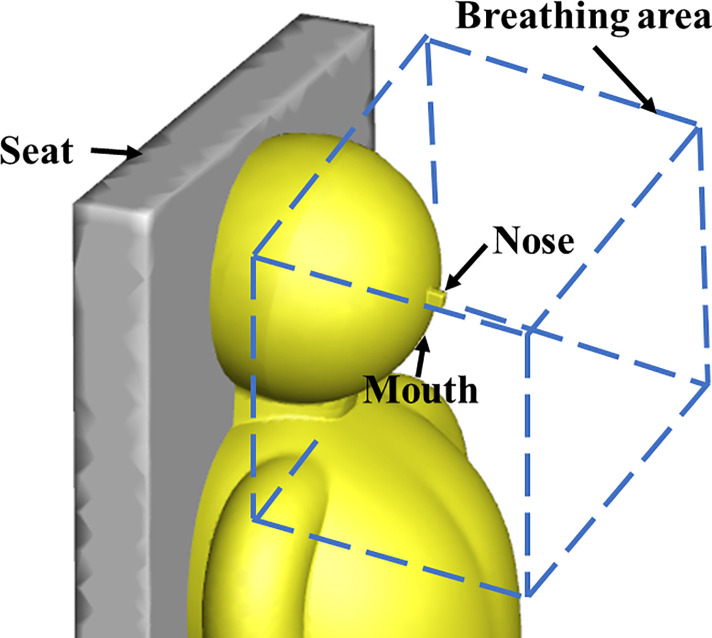
Passenger breathing area.

In the current study, the droplet dispersion was investigated in a real bus with a dimension of 11.5 m × 2.5 m × 2 m (L × W × H). The arrangement of seats inside the bus is shown in Fig. 1. The driver's seat and 12 rows of passenger seats were on the left side, and 11 rows of passenger seats were on the right side. Figure 2 shows the layout of the air supply diffusers. There were two round air supply diffusers with a diameter of 0.05 m along the air duct, 16 air supply diffusers with a diameter of 0.05 m at the head and tail of the cabin, and seven rectangular air supply diffusers evenly distributed above the luggage rack on both sides of the cabin, with dimensions of 0.13 m × 0.037 m (L × W). The total area of the air supply diffusers was 0.22 m^2^. The return vent was located on the ceiling and had dimensions of 0.65 m × 0.44 m (L × W). The human body was simplified and made up of 5 parts: head, body, arms, legs, and feet. The height for all passengers was assumed to be 170 cm. Passengers were also assumed to wear summer clothes. As shown in Fig. 3, the breathing area was a cube with a volume of 0.027 m^3^. The center of the cube was at the nose.

**FIG. 4.**
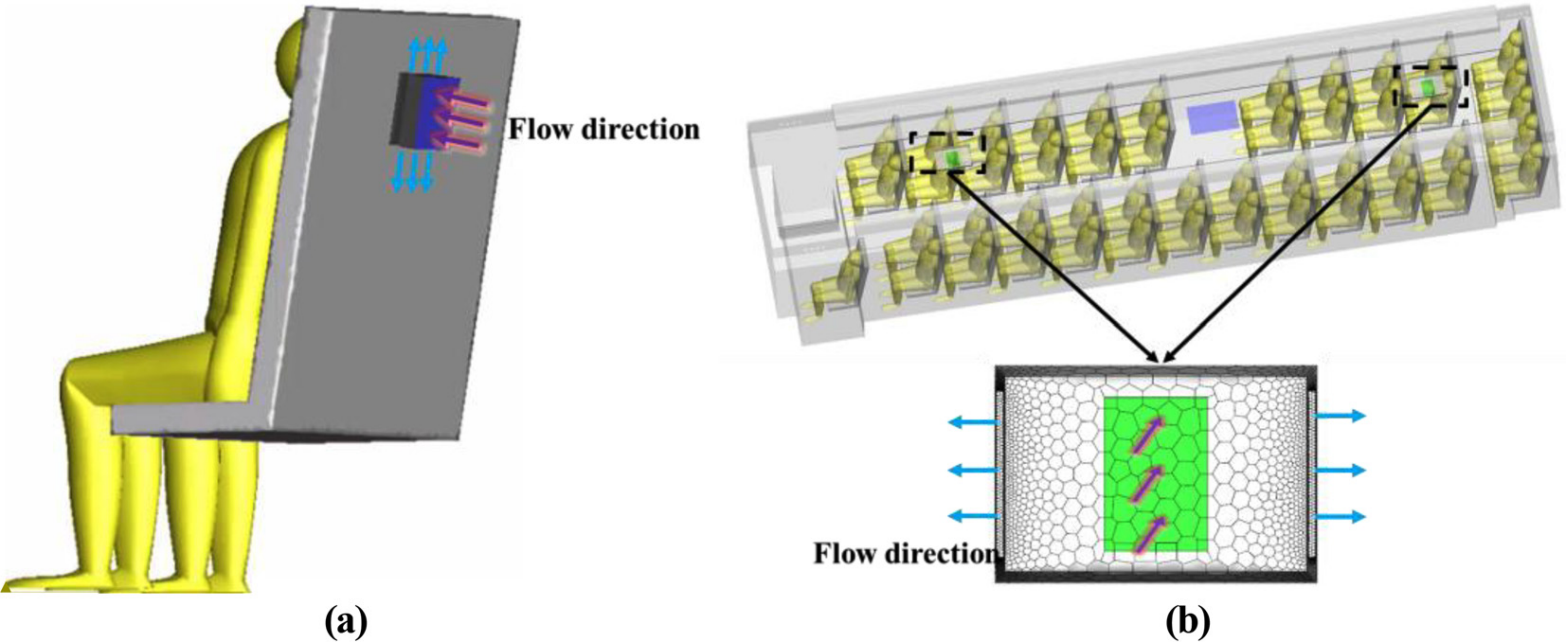
Location of the purifier (a) one purifier on seat back and (b) two large purifiers on ceiling.

Four different-sized droplets (1 *μ*m, 5 *μ*m, 10 *μ*m, and 20 *μ*m) were considered in the investigation. Previous studies indicated that droplets of the above sizes are most easily inhaled by humans.^35–37^ Three different infected passengers (P7, P21, and P46) were selected to study the effect of the location of the droplet source on the dispersion characteristics of droplets in the bus. In addition, two different types of purifiers were examined in the current study. As shown in Fig. 4, one was a small purifier installed on each seat back (with a flow rate of 18 m^3^/h), and the other consisted of two large purifiers installed on the ceiling at the front and rear of the bus (with a flow rate of 200 m^3^/h). To further study the effect of purifiers on droplet removal in different locations, P7 and P46 were considered to be infected passengers. Furthermore, all details about the case setup are outlined in Table I.

**TABLE I.** Case descriptions.

Investigation	Infected passenger	Particle size (*μ*m)	purifier	
Effect of particle size	P7	1	No	CASE1
	P7	5	No	CASE2
	P7	10	No	CASE3
	P7	20	No	CASE4
Effect of infected passenger location	P7	5	No	CASE5
	P 21	5	No	CASE6
	P46	5	No	CASE7
Effect of purifier	P7	5	Small purifier	CASE8
	P46	5	Small purifier	CASE9
	P7	5	Large purifier	CASE10
	P46	5	Large purifier	CASE11

**FIG. 5.**
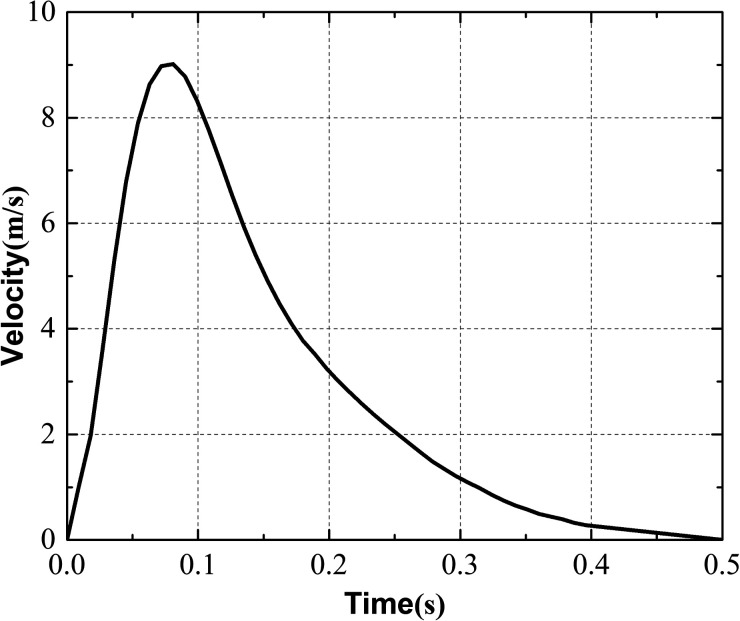
Airflow velocity of a single cough over time.

The heat flux of the body surface was assumed to be 20 W/m^2^, whereas the heat transfer coefficient of the ceiling and floor was 3 W/(m^2^·K), the heat transfer coefficient of the windows was 5 W/(m^2^·K), and the seats and other boundaries were adiabatic. The exhaled airflow induced by coughing of the infected passenger was modeled using a UDF. As shown in Fig. 5, the duration of a single cough was 0.5 s, and the maximum velocity was approximately 9 m/s.^20,38,39^ What's more, the angle between the exhaled airflow and the horizontal plane was 30 degrees, and to obtain a stable distribution of the expiratory droplets, one hundred thousand droplets with a density of 980 kg/m^3^ were released during a single cough. Evaporation was neglected. For interactions between droplets and different surfaces, the trap condition was imposed on the solid walls, and an escape condition was imposed for the air supply diffuser and vents. Droplets captured by the purifier would be removed from the fluid domain, and the UDF was applied to achieve this function. The UDF also recorded the number of droplets deposited on different surfaces and the number of droplets removed by the different purifiers. The details of the boundary conditions and solver settings are listed in Table II. All simulations were performed using the commercial CFD software FLUENT 19.2. For numerical solutions, a second-order upwind discretization method was used for convective terms, and the Semi-Implicit Method for Pressure Linked Equations (SIMPLE) algorithm was adopted to couple pressure and velocity.

**TABLE II.** Boundary conditions settings.

Boundary	Setting
Air supply diffuser	Velocity: 3.5 m/s, perpendicular to the diffuser, temperature: 290 K, turbulent intensity: 10%, escape.
Vent	Pressure outlet, 0 Pa, escape.
Body surfaces	No slip; heat flux: 20 W/m^2^, trap.
Ceiling, floor, sidewall	Heat transfer coefficient: 3 W/(m^2^·K); trap.
Windows	Heat transfer coefficient: 5 W/(m^2^·K); trap.
Seats	Adiabatic; trap.
Mouth (infected passenger)	Velocity various with time (user define function); trap.

In order to determine the appropriate grid strategy, three grid resolutions (4.6 million, 9.2 million, and 12 million) were tested for grid independence by case 1, and the average velocity of the airflow at the three cross sections surface was used as the evaluation index. As shown in Table III, the difference between the results of 9.2 million and 12 million grids is less than 5%. Hence, all the following computations are based on the 9.2 million grids.

**TABLE III.** Grid independence verification.

X-directional cross section	Velocity (m/s)		
	4.6 million	9.2 million	12 million
1.5 m	0.263	0.252	0.256
4.5 m	0.256	0.265	0.271
8.5 m	0.268	0.277	0.265

**FIG. 6.**
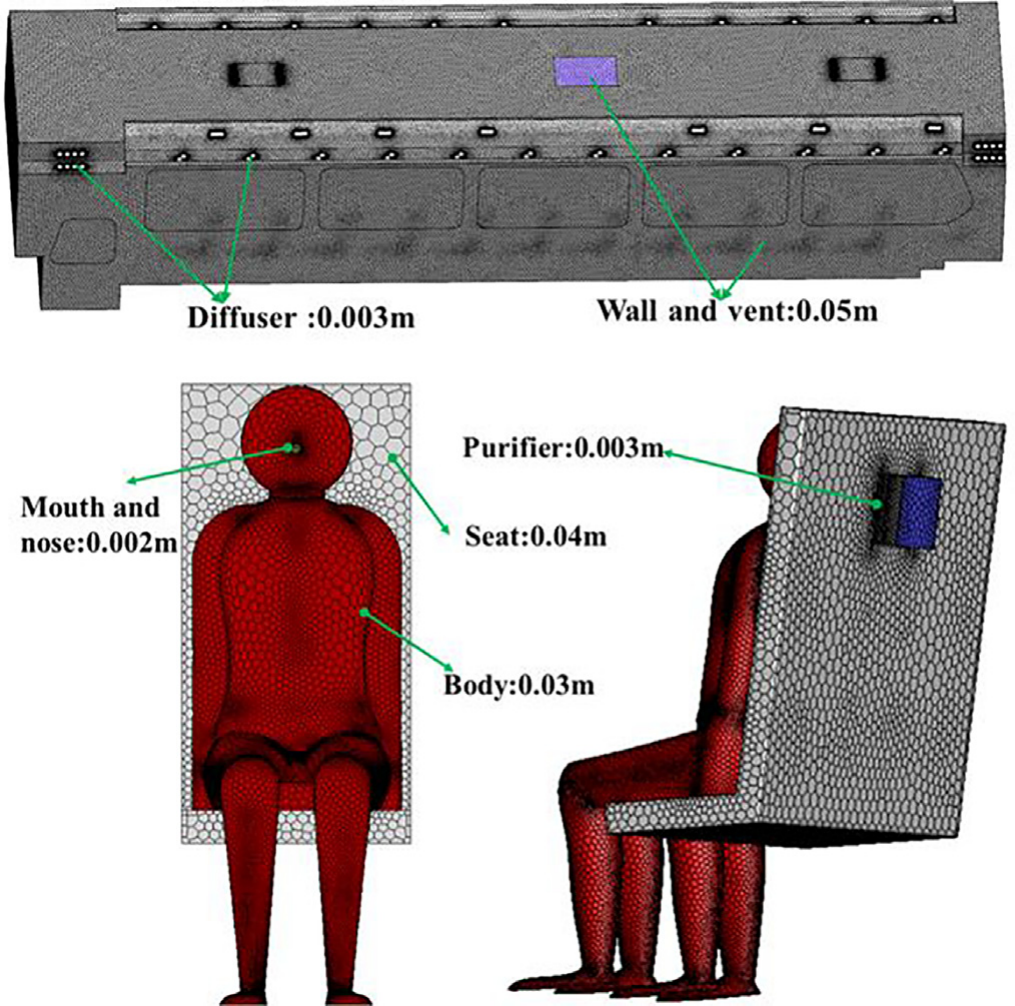
Grid resolution in the bus.

For all simulation cases, unstructured meshes were generated using Fluent meshing19.2. As shown in Fig. 6. The grid consisted of 2 mm for the mouth, nose, and face of the passengers, 20 mm for the rest of the body of the passenger and seats, 3 mm for the air supply diffusers and purifiers, and 40 mm for others. The skewness of more than 99.5% of the cells is less than 0.88.

### B. Validation

**FIG. 7.**
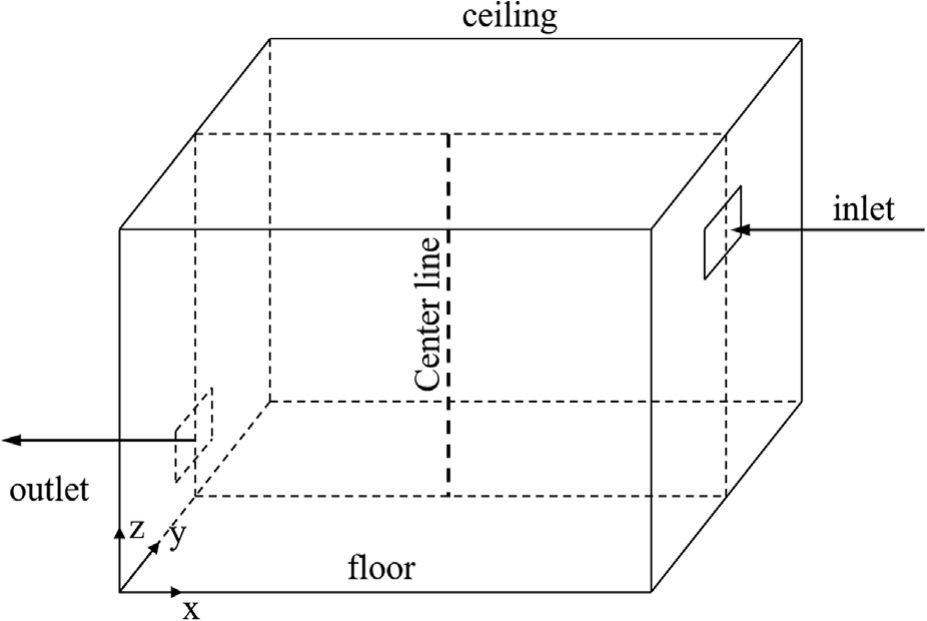
Schematic illustration of the test chamber.

**FIG. 8.**
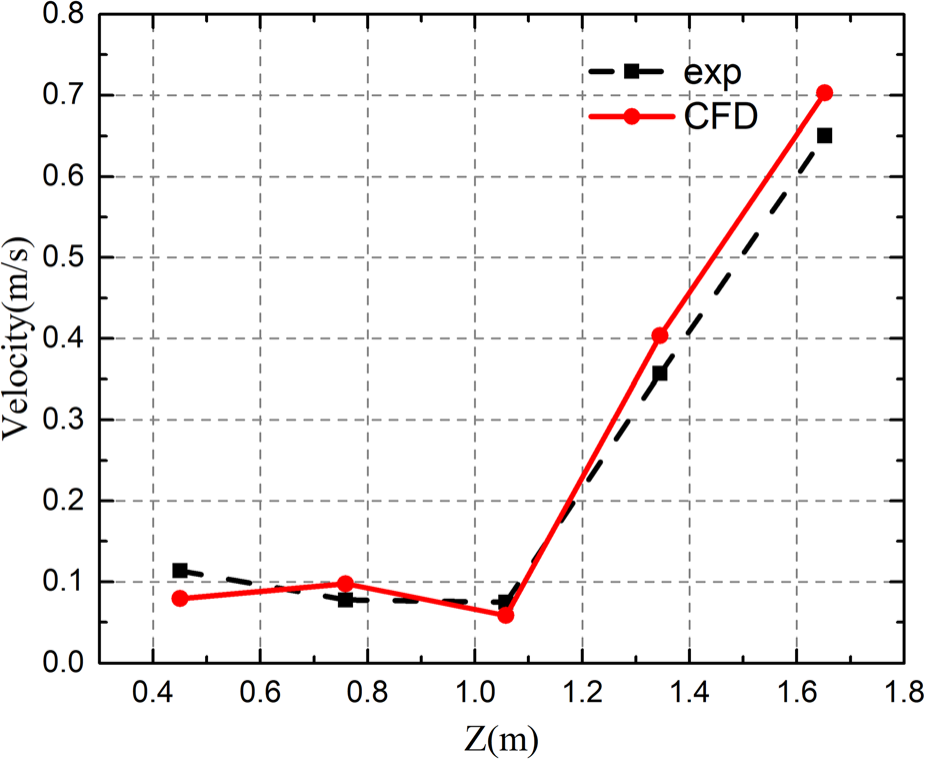
Velocity magnitude at the centerline of the chamber.

The present numerical model for simulating the airflow field was validated based on the experimental data of Zhang *et al.*^40^ Figure 7 shows the chamber geometry (L × H × W = 4 m × 2.4 m × 2.1 m), and the test chamber had an inlet and an outlet. Figure 8 shows the airflow velocity profile along the centerline of the chamber. It can be seen that the simulation results agreed well with the experimental data.

## IV. RESULTS AND DISCUSSION

The dispersion of droplets originating from a single cough by the infected passenger was modeled. In order to conserve computing resources, the calculation was terminated when the suspended droplets in the bus contained <1% of the total released droplets. The simulation results for the different cases are presented and discussed in this section. The simulation results present the flow field and characteristics of droplet dispersion in the cabin with and without the purifier, and the effect of droplet size, source location, and purifier type were taken into consideration. The deposition of droplets on different surfaces, the deposition rate, and the performance of purifiers in removing droplets were analyzed. In addition, the Wells–Riley equation was used to assess the passenger infection risk.

### A. Cases without purifier

#### 1. Flow field inside the bus

**FIG. 9.**
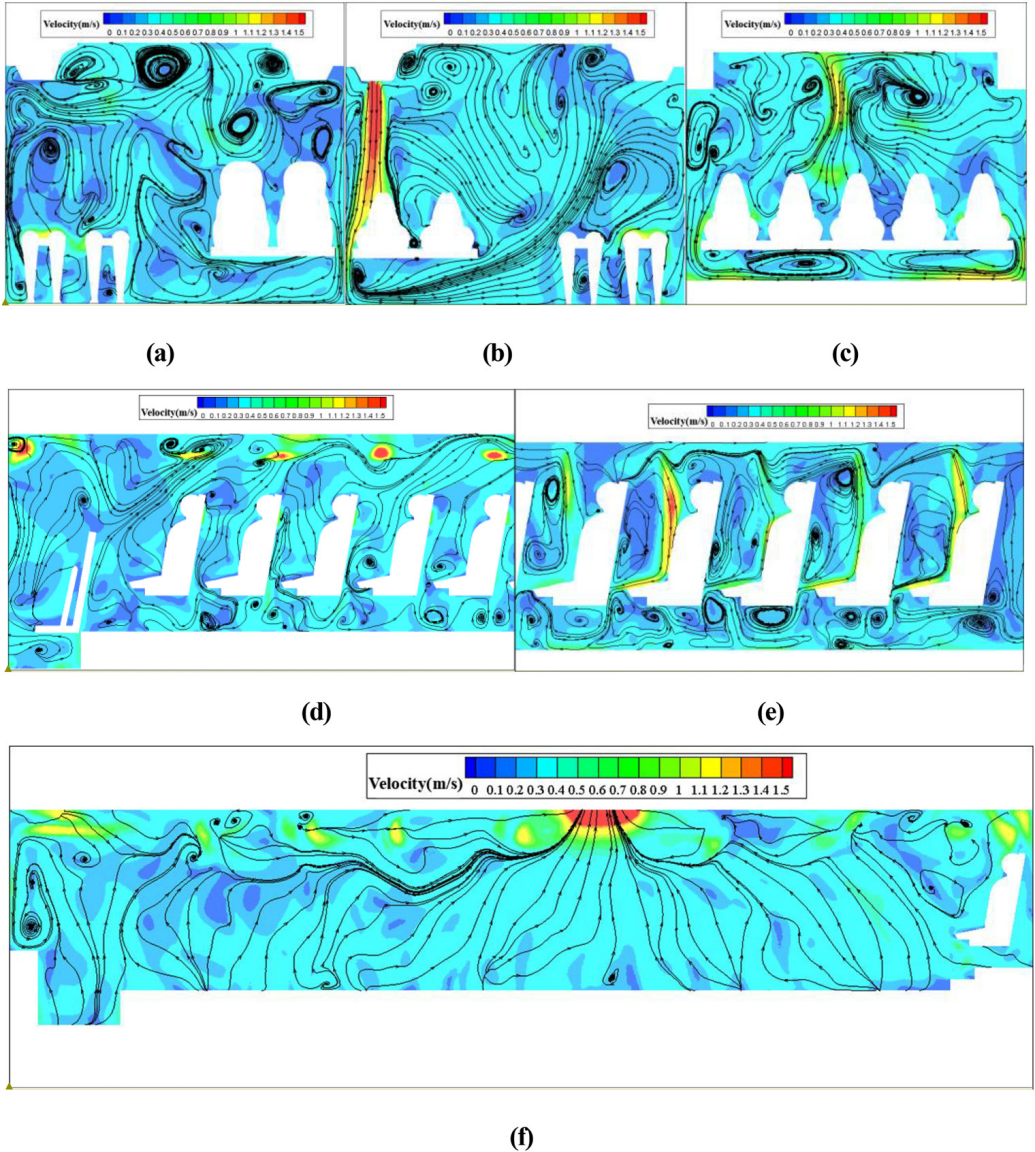
Velocity streamlines in the cabin on cross-section at (a) x = 1.2 m, (b) x = 4 m, (c) x = 8.9 m, and longitudinal section through (d) y = −0.46 m, (e) y = 0.46 m, and (f) y = 0 m.

**FIG. 10.**
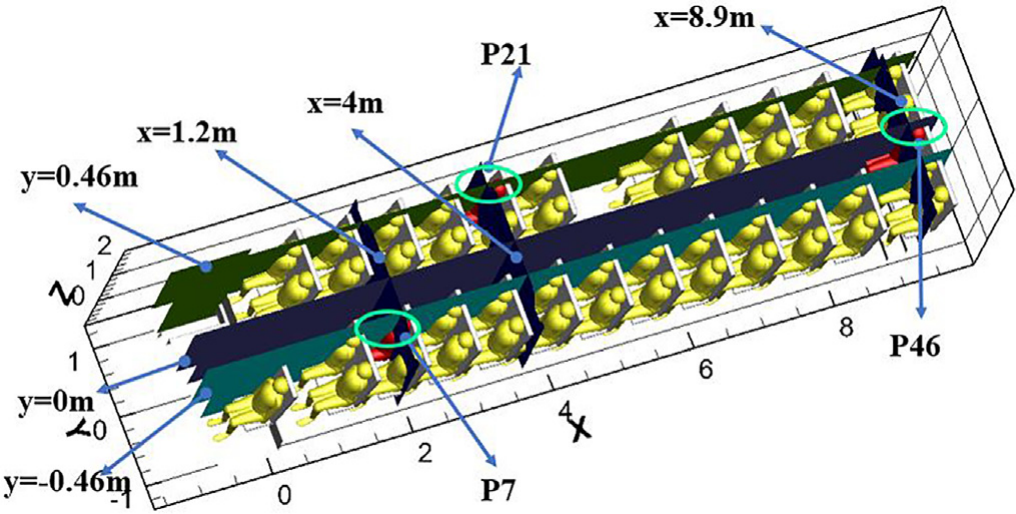
Selected planes of x = 1.2 m, 4 m, and 8.9 m (in front of P7, P21, and P46, respectively), y = −0.46 m, 0 m, and 0.46 m (through P7, P21, and P46, respectively).

To accurately calculate the spread of coughed droplets, it is necessary to use the steady-state flow field as the initial condition of the simulation. Figures 9(a)–9(c) show the streamlines in a cross-section in front of P7, P21, and P46, and Figs. 9(d)–9(f) show the streamlines in the longitudinal section through P7, P21, and P46, respectively (Fig. 10 shows the location of these sections). Cold air from the air supply diffuser flowed downward vertically. A portion of the cold air was obstructed by the passengers and seats, and then, a clockwise vortex was formed in the zone between the passenger and the seat. The remaining cold air flowed toward the aisle from beneath the seat, creating large re-circulations in the cross section. The airflow in the upper part of the cabin flowed backward due to the vent located at the top and the middle of the bus above the seventh-row seats. In addition, the presence of small and large vortices on both sides of the upper part of the cross-section indicated that the airflow consisted of strongly mixed convection in the cross section.

As shown in Figs. 9(a)–9(c), the flow field structure was different in the three cross sections. One notable difference compared with the cross sections at x = 4 m was that more airflow from the air supply diffusers on the left side flowed to the right side in the cross-section at x = 1.2 m and x = 8.9 m. This can be attributed to the asymmetric arrangement of the air supply diffusers. As shown in Figs. 9(d)–9(f), longitudinal airflow dominated in the cabin due to the presence of the vent, and the airflow then moved upward along the passengers' bodies to the upper part of the cabin due to the seat-blocking effect and thermal plumes around the passengers' bodies and then quickly flowed to the vent.

**FIG. 11.**
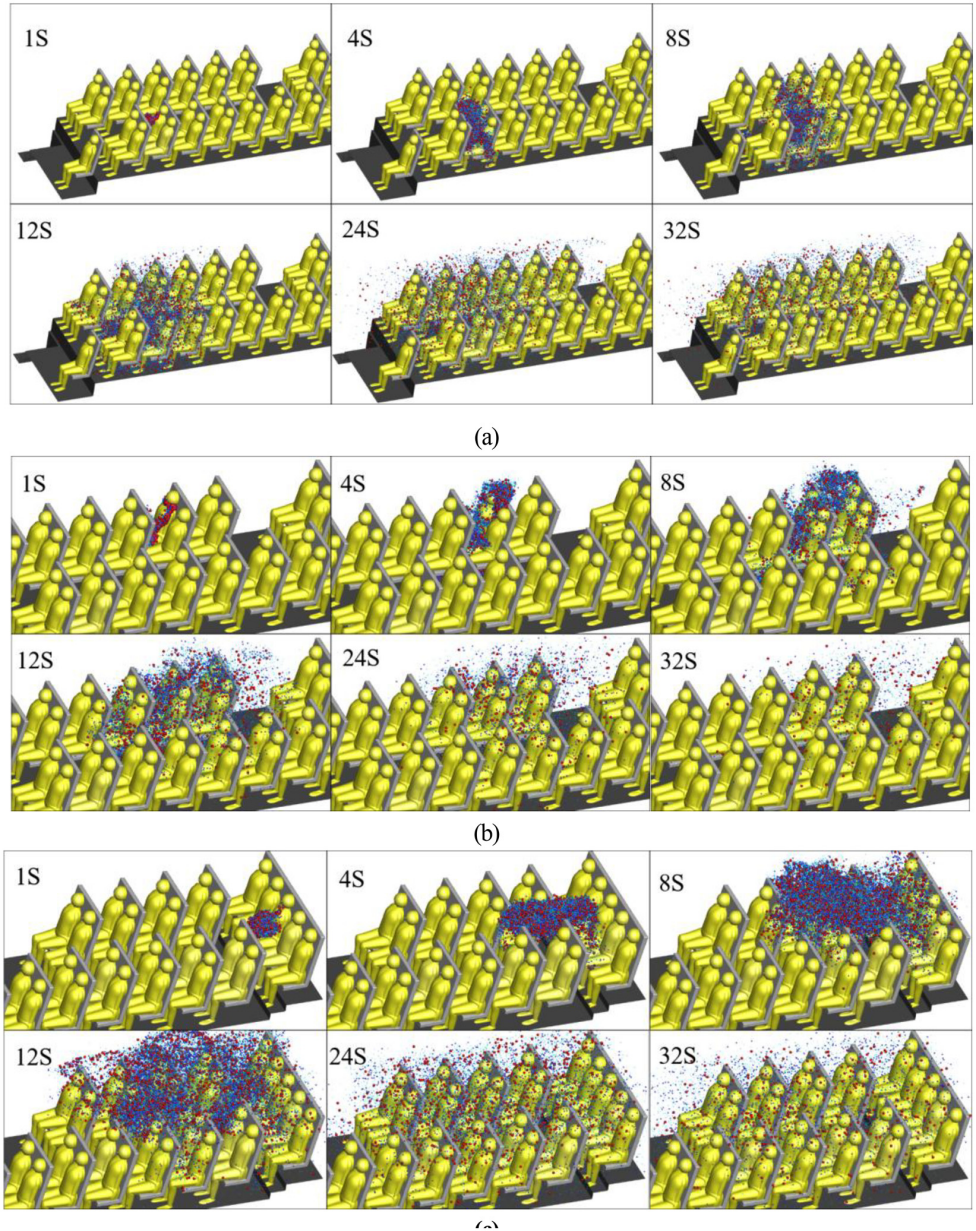
Distribution of aerosol particles in the bus at different time for the (a) P 7, (b) P21, and (c) P46.

The transport of respiratory droplets generated by coughing is a transitory process. For the purpose of describing the dispersion characteristics of droplets, a single-release impulse source was used. Figures 11(a)–11(c) illustrate the transport of coughed droplets exhaled by P7, P21, and P46. After 1 s of coughing, droplets first followed the jet of air caused by the cough, which pushed the droplets down and forward. The air jet carried the droplets and traveled 0.45 m axially. The coughed droplets then followed the bulk airflow. There were two patterns of droplet spread, one in which the droplets were captured by the lateral airflow and moved to P6, and another in which a small proportion of the droplets moved to the upper part of the cabin due to the clockwise vortex or air flow. Finally, all of the droplets rose toward the ceiling due to the upward airflow continuing to move backward across the seats and passengers. The droplets moved toward the vent rapidly and exited once they reached the upper part of the cabin. It should be noted that only a small proportion of the droplets diffused to the right side of the bus.

When the infected passenger was P21, most of the droplets followed the downward airflow from the air supply diffuser and were blocked by passengers and deposited. Some droplets moved across P21 and arrived in the breathing zone of P25. When the infected passenger was P46, most of the droplets were carried by the strong airflow as shown in Fig. 9(f). Then, as mentioned above, the droplets moved toward the vent and exited the cabin. The vortices on both sides of the cabin captured a small proportion of the droplets, causing them to diffuse around the area in front of the last row.

#### 2. Effect of droplet size

**FIG. 12.**
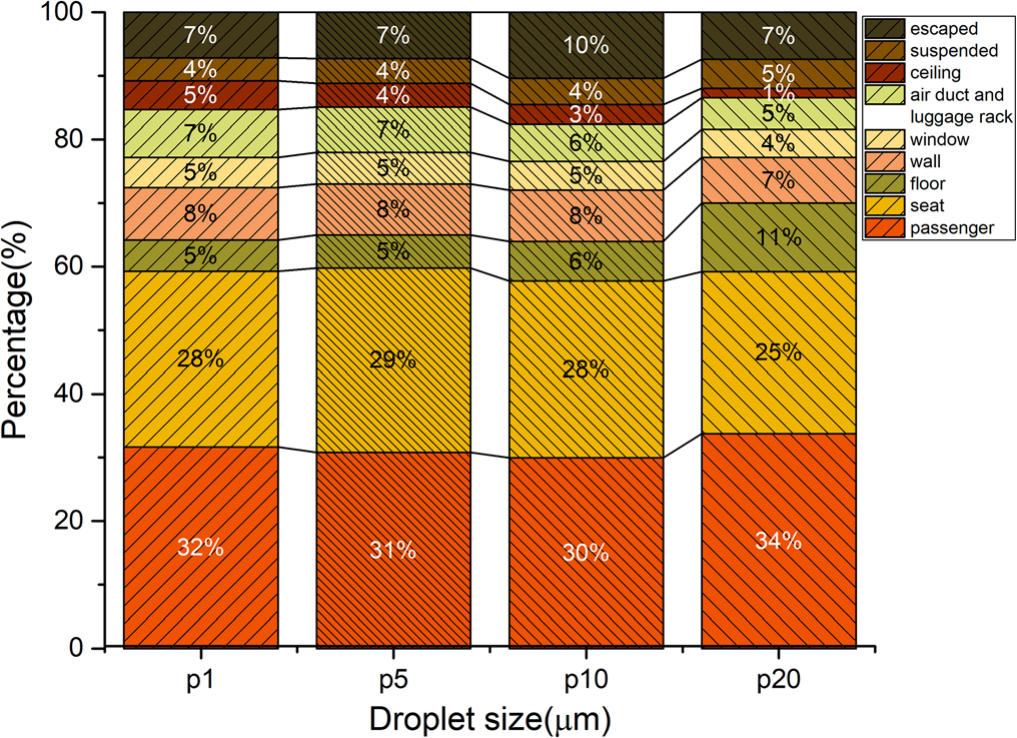
Effect of droplet size on deposition.

Droplet size is an essential factor in the dispersion process. Figure 12 shows the effect of droplet size on the deposition fraction on different surfaces in the bus. Droplets with a size of 1 *μ*m, 5 *μ*m, 10 *μ*m, and 20 *μ*m exhibited a similar deposition distribution on different surfaces. More than 60% of the deposition of droplets of different sizes could be attributed to obstruction of the airflow by seats and passengers. The deposition fraction on seats, passengers, and the floor in the case of 20-*μ*m droplets was 20% greater than that of 5-*μ*m droplets, and the deposition fraction on the upper structures of the bus (ceiling, air ducts, and luggage rack) was only 6%.

**FIG. 13.**
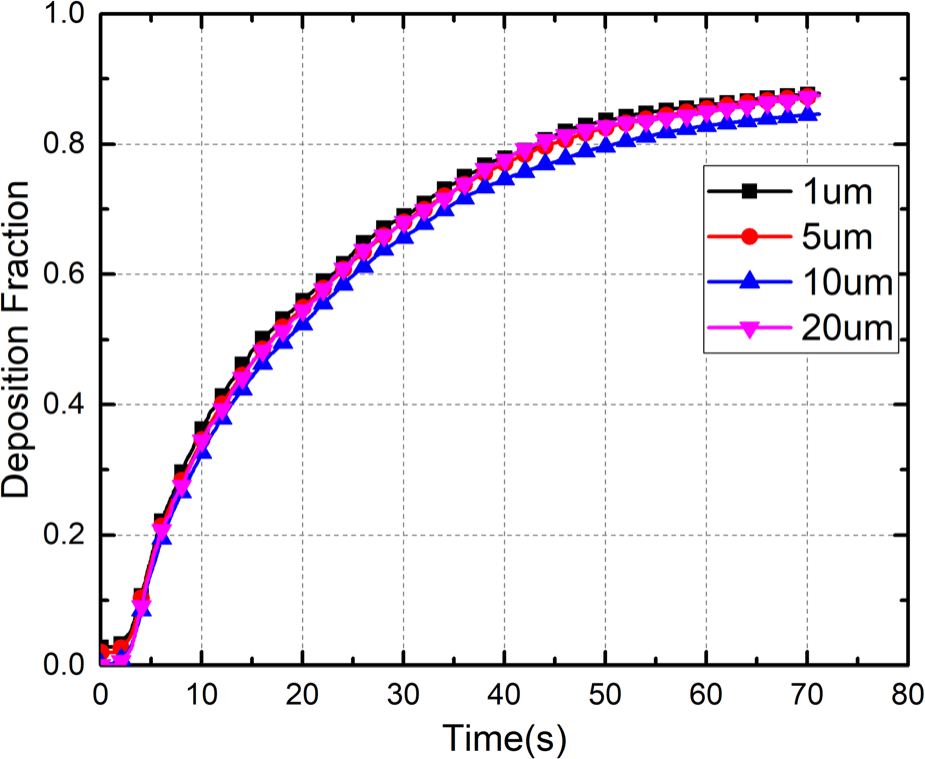
Deposition fraction of droplets with different size at different times.

Figure 13 shows the effect of droplet size on the deposition rate in the bus. Deposition can reduce the number of droplets in the air by 60% within 1 min. The deposition rates of droplets of different size were almost identical because of the high occupant density and confined space in the bus. These observations suggest that gravity does not play a significant role in transport of droplets in the size range of 1–20 *μ*m. In the current study, it was assumed that the droplets would be captured once they interacted with a surface, which could have led to overestimation of the droplet deposition rate. However, the airflow velocity in most regions of the cabin was quite low (0.1–0.3 m/s), such that the droplets would not have enough energy to overcome the van der Walls forces after colliding with a surface.^41,42^ Therefore, this assumption was reasonable.

#### 3. Effect of infected passenger location

It is evident that the location of the infected passenger significantly affects the trajectory of cough-generated droplets. Therefore, analyses of the effect of the location of the infected passenger on the fate of coughed particles would be relevant for elucidating the virus transmission mechanism. Hence, the transport of 5-*μ*m droplets exhaled from a single cough by an infected passenger in the front, middle, and back of the cabin (P7, P21, and P46, respectively) was investigated using the Lagrangian method.

**FIG. 14.**
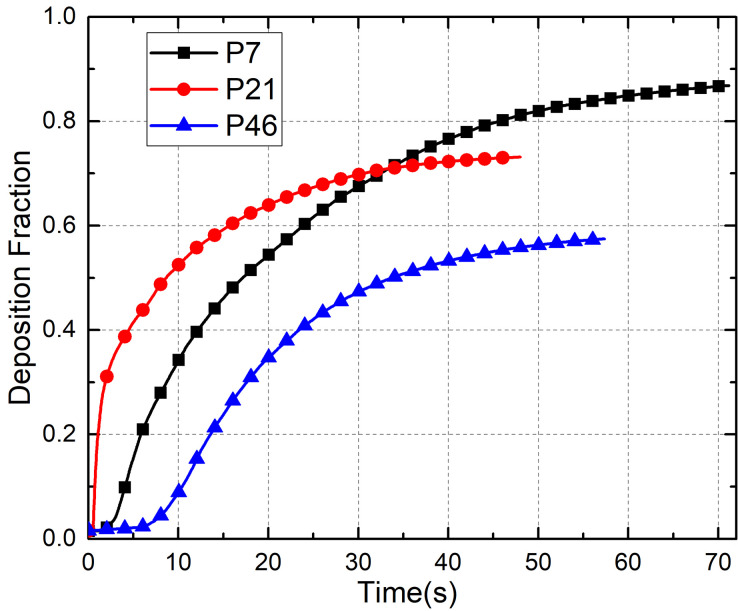
Deposition fraction of 5-*μ*m droplets from different infected passenger locations at different times.

The results of these analyses are shown in Fig. 14, which illustrates the deposition fraction of 5-*μ*m droplets generated in different locations in the bus. The deposition rate of droplets in the case of P21 was the highest among the three cases. This result can be explained by the droplets following the downward airflow from the air supply diffuser and then being blocked by the passenger's body, such that almost 20% of the droplets are deposited on P21. The lowest deposition rate of droplets was observed in the case of P46, as there was no obstruction of seats at the beginning of release. In addition, the fraction of droplets that exited through the vent was >20% except in the case of P7.

**FIG. 15.**
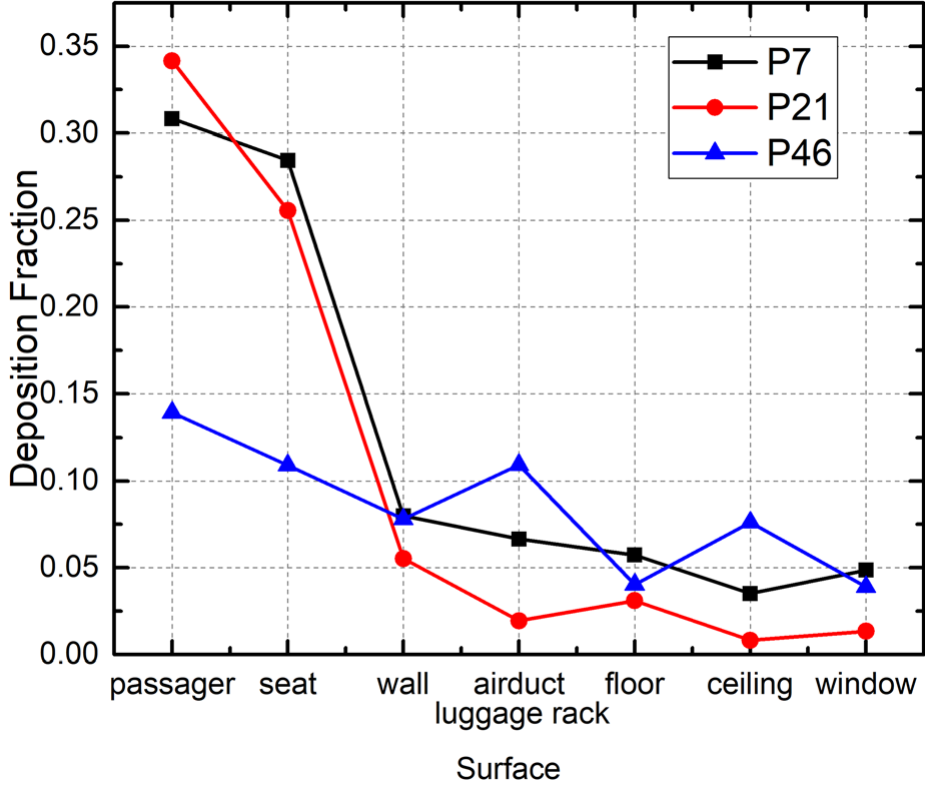
Effect of infected passenger location on droplet deposition on different surfaces.

Figure 15 shows the deposition fraction on the different surfaces in the bus for each of the three cases. The deposition distribution in the case of P7 was similar to that in the case of P21. The droplet deposition fraction on the passengers and seats was >60%, whereas it was <5% on the floor and only 5%–10% on the upper surfaces of the bus (ceiling, air ducts, and luggage rack). The droplet deposition fraction in the case of P21 was smaller because more of the droplets were discharged from the bus. With regard to P46, the deposition on the upper surfaces of the bus increased to 18% but decreased to 23% on the seats and passengers. This difference could be attributed to the higher position of the source location (the last row of seats is elevated relative to the other rows).

### B. Cases with purifiers

#### 1. Effect of the purifier: Impact on airflow streamlines and droplets dispersion

**FIG. 16.**
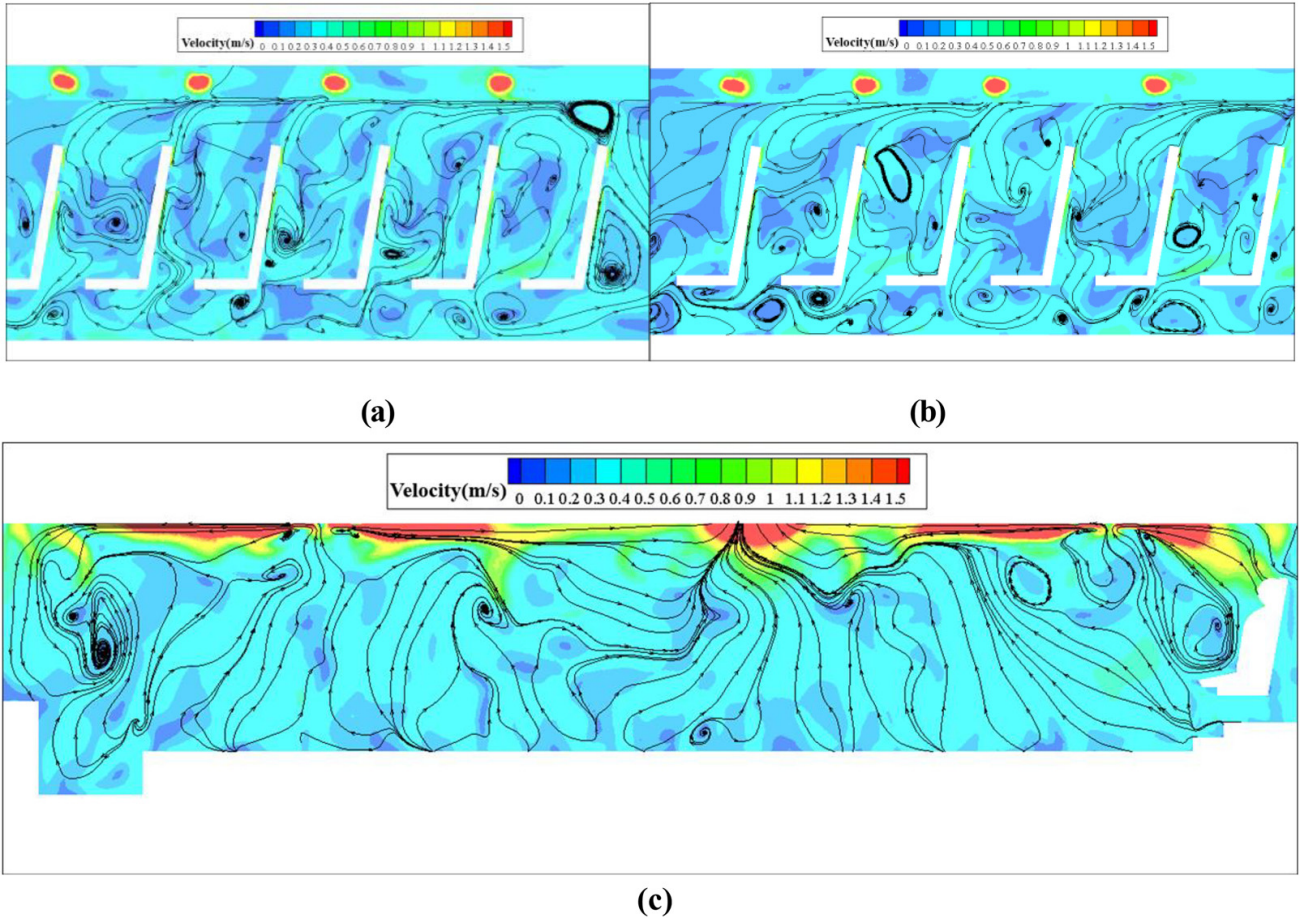
Velocity streamlines in the cabin on the longitudinal section through (a) y = −0.7 m, (b) y = 0.7 m, (small purifier) and (c) y = 0 m (large purifier).

**FIG. 17.**
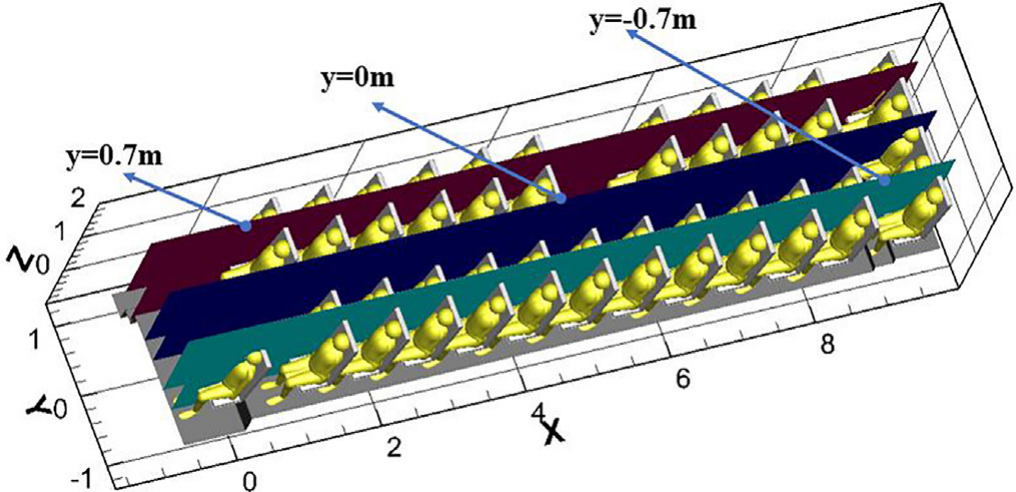
Selected planes of y = −0.7, 0.7, 0 (through small purifier, P46, respectively).

**FIG. 18.**
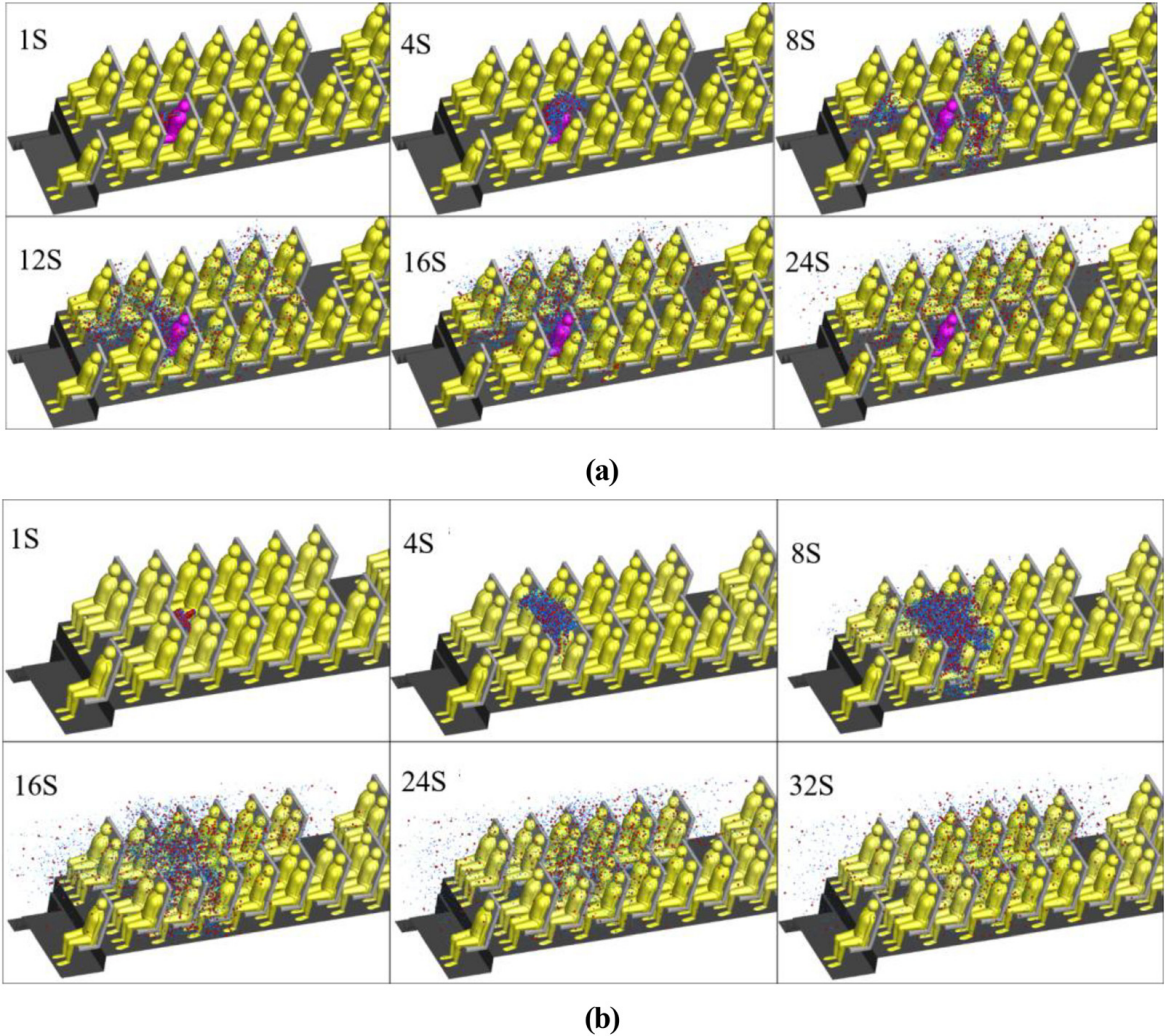
Temporal distributions of droplets due to a single cough by passenger 7 in the case of (a) small purifier and (b) large purifier.

Figures 16(a) and 16(b) show the air streamlines in the longitudinal section at y = −0.7 and y = 0.7 (through the small purifier, and Fig. 17 shows the location of longitudinal sections). The air between the seats was drawn by the small air purifier and then sent partly toward the upper part of the cabin and partly toward the floor. Finally, two small vortices in the upper and lower parts of the zone between the passenger and the front seat were formed. As a result, the released droplets were driven by the airflow from the purifier upward and across the seats in part and then moved downward and toward the floor in part [Fig. 18(a)].

Figure 16(c) shows the air streamlines in the longitudinal section and through the aisle in the case of the large purifier. The two purifiers installed on the ceiling altered the flow field significantly. Part of the air in the front and rear of the cabin flowed to the purifier and was sent out at a relative high velocity (>1 m/s). Such airflow patterns led to droplet diffusion [Fig. 18(b)]. There were clearly more droplets in the upper part of the cabin than in the case with the small purifier. This difference can be attributed to the negative pressure effects of the large ceiling-mounted purifier.

#### 2. Effect of the purifier: Impact on droplets deposition

**FIG. 19.**
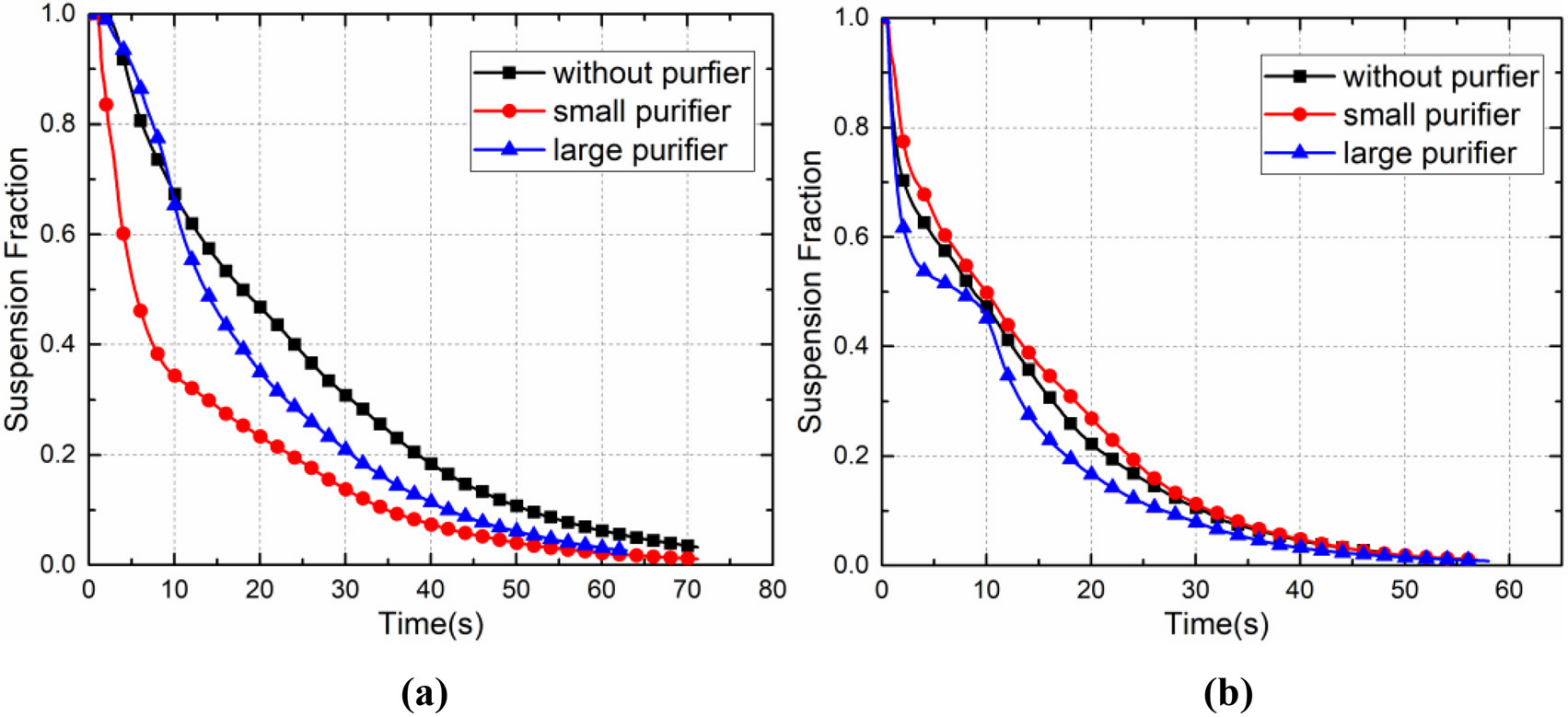
Temporal development of the suspension fraction of droplets in the case of (a) P7 and (b) P46.

As shown in Fig. 19, the two types of purifiers performed well in removing droplets generated at the front and rear of the bus, and the effectiveness of the purifiers in removing droplets was directly related to the location of the infected passenger. The current study found that ceiling-mounted purifiers promote the upward movement of droplets, causing them to stay away from the breathing area of passengers. Ceiling-mounted purifiers are thus highly efficient at removing droplets generated in different areas of the bus. However, the small purifier induced a slight negative pressure effect, and its effectiveness at removing droplets was directly related to the location of the infected passenger.

**FIG. 20.**
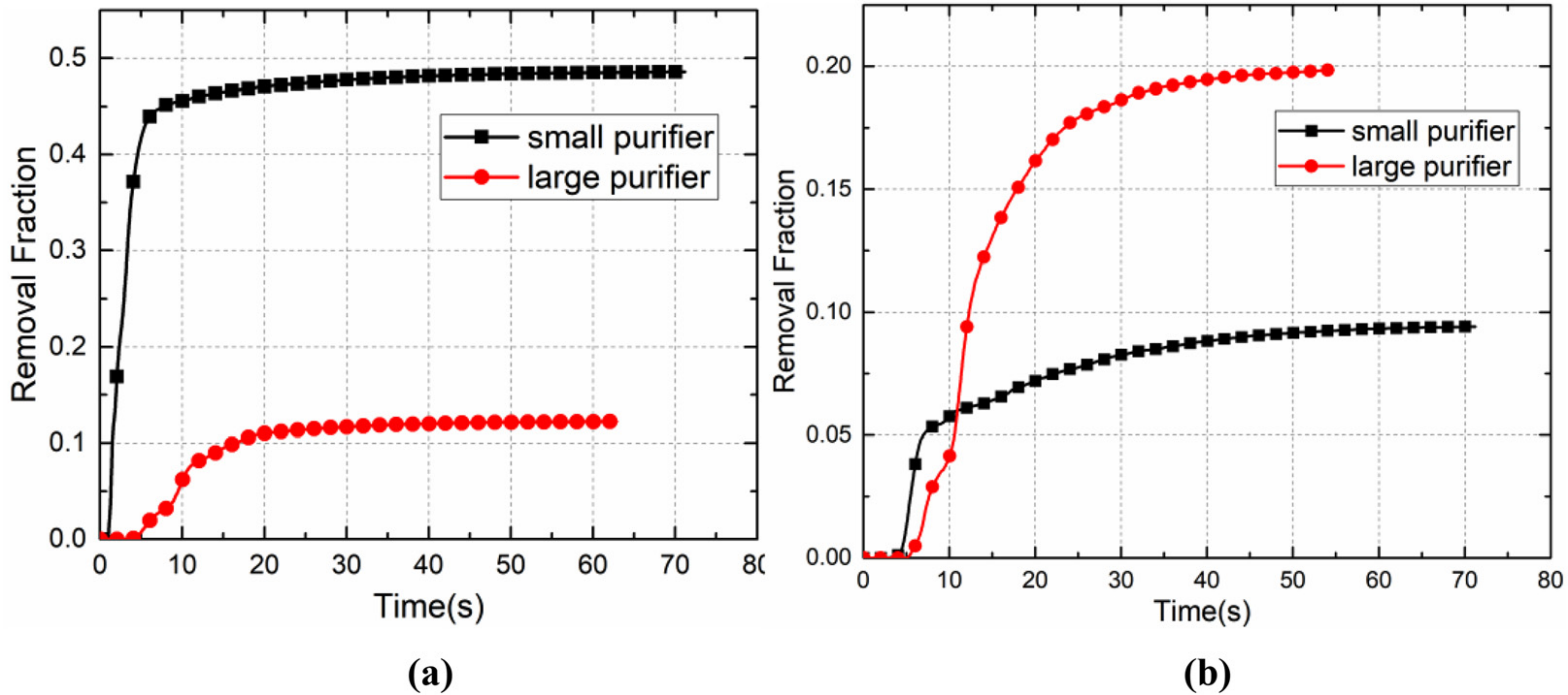
Temporal development of the removal fraction of droplets in the case of (a) P7 and (b) P46.

**FIG. 21.**
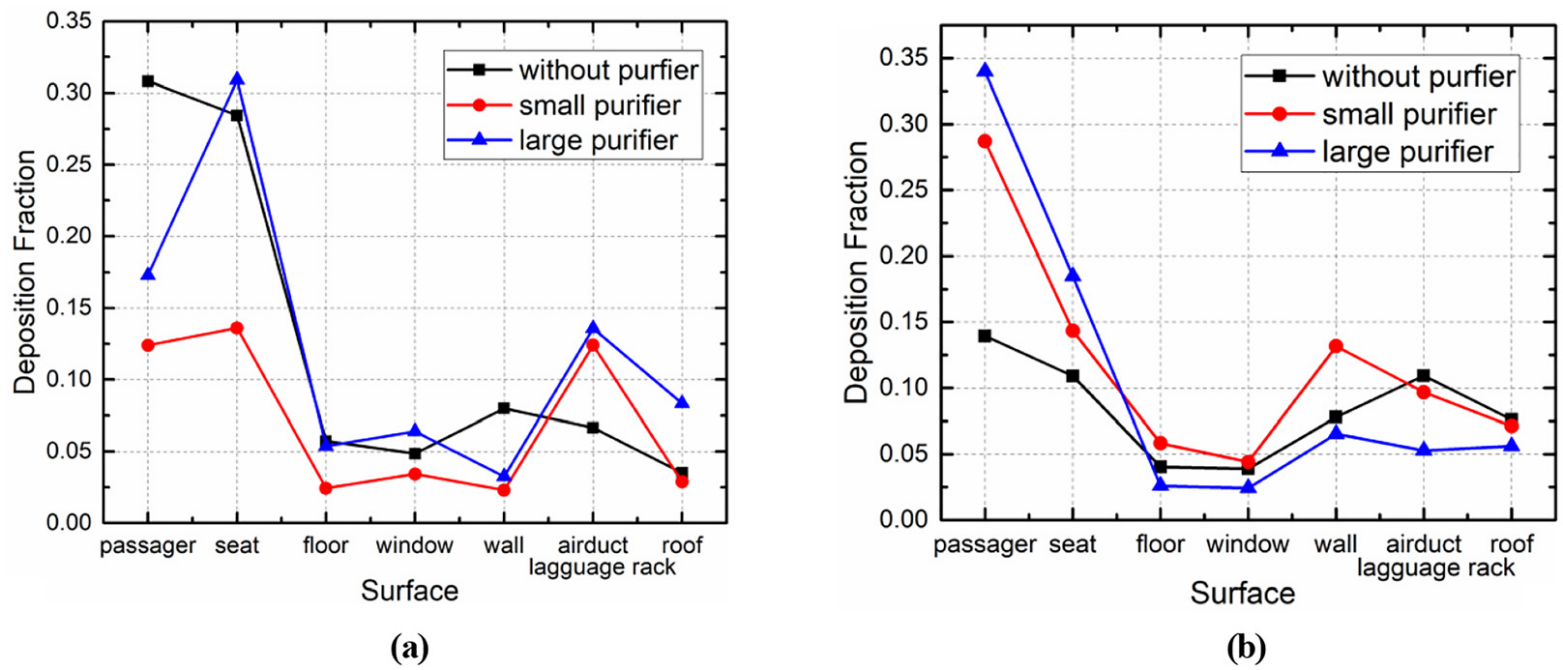
Droplet deposition on different surfaces in the case of (a) P7 and (b) P46.

As shown in Figs. 20 and 21, in the case of the small purifier with P7 as the infected passenger, the purifier was able to remove nearly 40% of the droplets in the first 5 s, while the other droplets continued to spread, with approximately 25% of the droplets deposited on the passengers and seats, 12% on the ceiling, air ducts, and luggage rack, 3% on the floor, and almost 3% of the droplets escaped from the cabin. In the case of the large purifier, the upward airflow was increased, such that the droplets moved faster to the upper part of the cabin, and 12% of the droplets were captured by the purifier, nearly 42% of the droplets were deposited on the passengers and seats, and 20% of the droplets were deposited on the ceiling and luggage rack.

In the case of P46 as the infected passenger, nearly 42% of the droplets were deposited on the passengers and seats. Most of the droplets spread toward the front and upward, driven by airflow quickly toward the vent. During this process, 15% of the droplets were deposited on the ceiling and luggage rack, nearly 8% of the droplets were captured by the purifier, and 20% of the droplets escaped from the cabin. In the case of the small purifier, the deposition fraction on the upper wall of the cabin was relatively large, which may have been caused by the upward airflow induced by the purifier. With the large purifier, 15% of the droplets were removed within 5 s after the cough, with approximately 55% of the droplets deposited on the passengers and seats, 12% on the ceiling, air ducts, and luggage rack, and nearly 15% of the droplets exited the cabin. The airflow generated by the large purifier drove the droplets toward the last row of passengers. As a result, more droplets were deposited on the passengers and seats in that area.

**FIG. 22.**
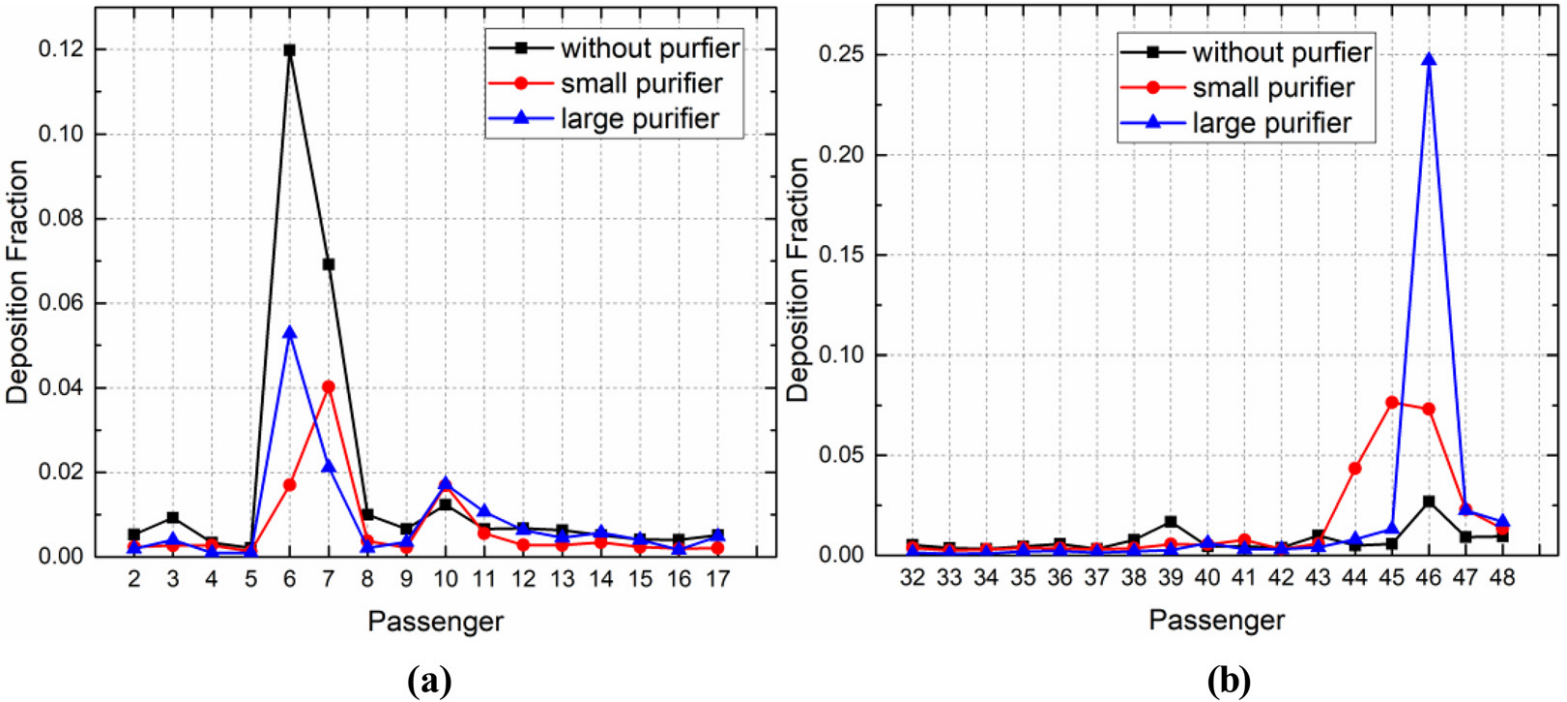
Deposition fraction on different passenger surface in the case of (a) P7 and (b) P46.

Figure 22 shows the distribution of deposition fraction on the body of passengers seated near the infected passenger. When the infected passenger was P7, compared with the case without a purifier, there were fewer droplets deposited on P6 in the case with the small purifier because almost 30% of the droplets were removed. The droplets produced by the infected passenger's cough moved upward across P6 and then backward. Some droplets were captured by the vortex between the seats and deposited on P10 and P11, and only a few droplets were deposited on P8, P9, P12, and P13, who were located on the other side of the cabin. The droplets deposited on the passengers in the case with the large purifier were smaller than in the case without a purifier, except for the droplets deposited on P10 and P11. This could have been due to the instability of the vortex between the seats.

With regard to P46 as the infected passenger, more droplets were deposited on P44 and P45. In the case of the small purifier, the flow field was asymmetrical in this region, as the air supply diffusers were essentially in a symmetrical arrangement. The flow field structure became unstable due to the turbulent fluctuation. The airflow could move to the left or to the right. In the current research, the droplets moved to the left due to the strong leftward flow. The deposition fraction on passengers increased to 21% in the case of the large purifier. This could be attributed to the airflow from the purifier driving the droplets toward the last row of passengers, causing more deposition on P46.

#### 3. Effect of the purifier: Impact on infection risk

**FIG. 23.**
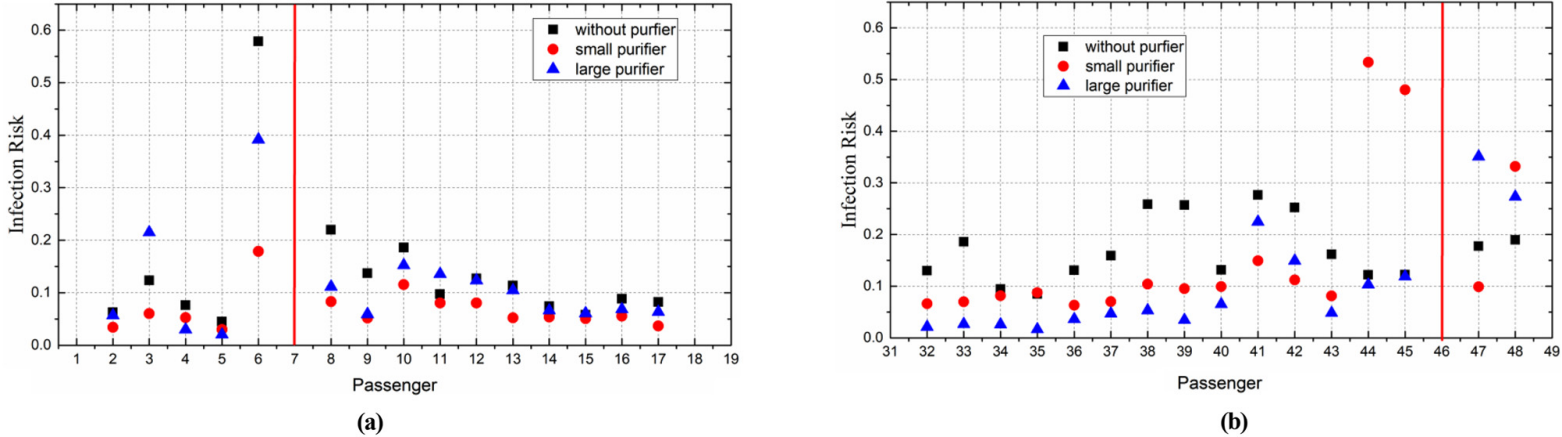
Infection risk of passenger in the case of (a) P7 and (b) P46.

Figure 23 shows the risk of infection for the passengers around the infected passengers after a 4-h trip, demonstrating the effectiveness of a purifier in reducing the infection risk of passengers. When the infected passenger was P7, the risk of infection was generally reduced (except for P11) in the case of the small purifier, perhaps because the airflow from the purifier caused more droplets to travel across the seat and into the breathing zone of P11. In the case of the large purifier, the risk of infection was effectively decreased except for P3. The increased risk of infection of P3 might have been caused by the droplets spreading into the breathing area carried by the airflow from the purifier.

When the infected passenger was P46, in the case of the small purifier, the droplets moved toward the left side in front of the last row of seats, such that the infection risk was increased for P44 and P45. The infection risk for P47 and P48 was increased in the cases of the large purifier because some of the droplets were carried back toward the last row of passengers by the airflow from the purifier, resulting in an increased risk to passengers in the last row.

## V. CONCLUSION

The purpose of the current study was to investigate the effect of small seat-mounted and large ceiling-mounted air purifiers on the removal of cough-generated droplets in buses and reducing the risk of infection of other passengers. The following conclusions can be drawn from the results:

(1) Purifiers can effectively remove droplets and, thus, reduce the risk of infection for almost 90% of passengers. Inside the cabin, a purifier can create areas of negative pressure that accelerate the removal of droplets, which can help to reduce the infection risk of most passengers.
(2) Airflow generated by the purifier will enhance droplet mixing, such that the droplets will be spread over a broader area compared with the absence of a purifier. However, the tendency for droplets to escape is inhibited. Therefore, the risk of infection for some specific passengers is increased. Our data suggest that the airflow generated by the purifier should have the same direction as the airflow in the original flow field.
(3) Ceiling-mounted large purifiers can significantly alter the structure of the flow field inside a bus, whereas small purifiers only affect the flow field around their location. Therefore, ceiling-mounted purifiers are highly efficient at removing droplets generated under a variety of conditions. In contrast, the performance of small purifiers in removing droplets is directly related to the location of the infected passenger.

Admittedly, some factors were not taken into account. For instance, the effect of air recirculation was ignored, which may cause higher probability to a passenger. The heat exchange between the wall and the environment was ignored, which may lead to inaccurate predictions of flow field near the wall. What is more, the removal efficiency of a purifier for droplets was assumed to be 100%. In fact, the removal efficiency for droplets is related to their particle sizes.

## SUPPLEMENTARY MATERIAL

See the supplementary material for the UDFs used in the current study.

## Data Availability

The data that support the findings of this study are available from the corresponding author upon reasonable request.
